# Card from Dr. J. F. Alexander

**Published:** 1897-05

**Authors:** J. F. Alexander

**Affiliations:** Atlanta


### CARD FROM DR. J. F. ALEXANDER.

ATLANTA, May 3, 1897.

*To the Board of Censors, Georgia Medical Association:*

I have just learned (for the first time) through the Atlanta *Constitution* that charges of unprofessional conduct were preferred against me before your Board at the late meeting of the Association, at Macon, and that I was found guilty of the same. This was done, as you doubtless know, without any notice to me, and without giving me an opportunity to be heard. It was charged that I had an interest in a hygienic institution. This was not true. I had no such interest, and had not had for four months prior to the meeting in Macon. *At any rate, I should have been allowed a statement or explanation.* I know that others whose "conduct" was investigated were notified of the charges and requested to appear before the Board in their defense. Was not the same courtesy due me?

Yours very truly,

J. F. ALEXANDER.

---

FOR hay fever a writer in the *American Medical Journal* says: Discard the use of sprays, and apply to the nostrils, on a cotton pledget, an unguent composed of six parts of cocaine muriate, ten of carbolic acid, twenty of menthol, one hundred and twenty of oil of sweet almonds, two hundred and forty of zinc ointment.

